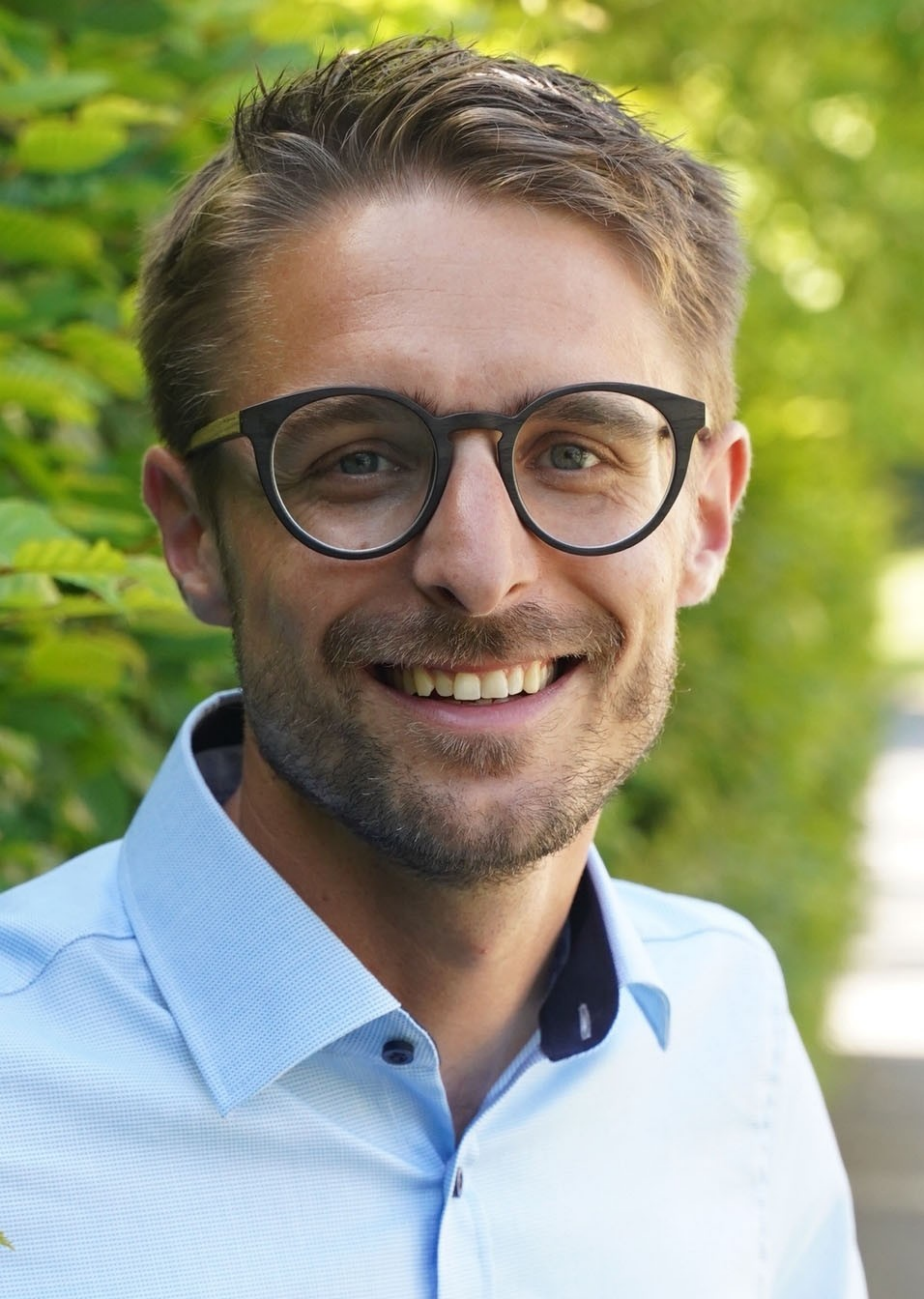# The Conversion of Carbon Monoxide and Carbon Dioxide by Nitrogenases

**DOI:** 10.1002/cbic.202100453

**Published:** 2021-11-05

**Authors:** Niels N. Oehlmann, Johannes G. Rebelein

**Affiliations:** ^1^ Max Planck Institute for Terrestrial Microbiology Karl-von-Frisch-Straße 10 35043 Marburg Germany

**Keywords:** C−C coupling, carbon dioxide, carbon monoxide, hydrocarbons, nitrogenases

## Abstract

Nitrogenases are the only known family of enzymes that catalyze the reduction of molecular nitrogen (N_2_) to ammonia (NH_3_). The N_2_ reduction drives biological nitrogen fixation and the global nitrogen cycle. Besides the conversion of N_2_, nitrogenases catalyze a whole range of other reductions, including the reduction of the small gaseous substrates carbon monoxide (CO) and carbon dioxide (CO_2_) to hydrocarbons. However, it remains an open question whether these ‘side reactivities’ play a role under environmental conditions. Nonetheless, these reactivities and particularly the formation of hydrocarbons have spurred the interest in nitrogenases for biotechnological applications. There are three different isozymes of nitrogenase: the molybdenum and the alternative vanadium and iron‐only nitrogenase. The isozymes differ in their metal content, structure, and substrate‐dependent activity, despite their homology. This minireview focuses on the conversion of CO and CO_2_ to methane and higher hydrocarbons and aims to specify the differences in activity between the three nitrogenase isozymes.

## Graphical abstract

**Nitrogenases** reduce inert dinitrogen to bioavailable ammonia and thus ‘feed’ the Earth's biosphere. To catalyze this challenging reaction, nitrogenases are equipped with a complex reduction machinery that also permits the remarkable conversion of carbon monoxide and carbon dioxide. In particular, the formation of methane and hydrocarbons opens up the prospect of future biotechnological processes for carbon capture and utilization by nitrogenases.

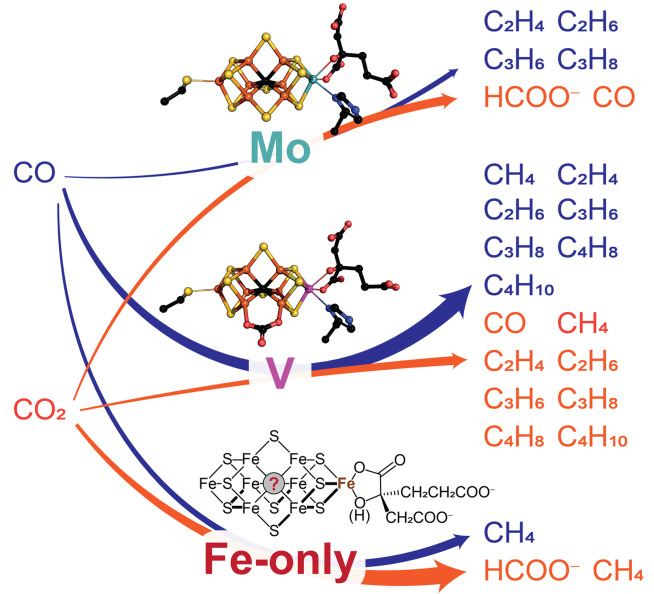

## 1 Introduction

### 1.1 Global nitrogen fixation

Bioavailable nitrogen (N) is essential for all life on Earth to build central metabolites such as nucleotides and amino acids.^[1]^ Although an inexhaustible supply of dinitrogen (N_2_) is stored in the Earth's atmosphere, N_2_ is inaccessible to most organisms due to its kinetic stability. The majority of bioavailable N is derived from the atmosphere through the reduction of N_2_ to ammonia (NH_3_) in a process known as nitrogen fixation. For millions of years, nitrogen fixation was unique to diazotrophic microorganisms.

With the beginning of the 20^th^ century, the industrial Haber–Bosch process was invented to reduce N_2_ to NH_3_, to produce explosives and fertilizers.^[2]^ The process requires the reactants to pass over a heterogeneous iron catalyst at >500 °C and 100 bar for the desired product formation.^[3]^ Due to the harmful effects of the process on the environment, it is imperative to work on a more sustainable N_2_ fixation. Remarkably, evolution produced a sophisticated enzymatic reduction machinery that is capable of reducing N_2_ under physiological conditions.^[4]^ Nitrogenases fix N_2_ by consuming ATP and low potential electrons with the following canonical molybdenum (Mo) nitrogenase stoichiometry [Eq. 1]:

(1) $$N{}_{2}+8H{}^{+}+16\mathrm{ATP}+8e{}^{-}\to 2\mathrm{NH}{}_{3}+H{}_{2}+16\mathrm{ADP}+16P{}_{i}$$

In contrast to the Haber−Bosch process, nitrogenases rely on reducing equivalents stored in flavodoxins or ferredoxins instead of electrons from elemental hydrogen (H_2_); nitrogenases rather produce H_2_ as byproduct. Additionally, nitrogenases require hydrolysis of ATP for their electron transfer.^[5]^ The N_2_ fixation by nitrogenases reaches a reaction rate of ∼1 s^−1^ which is a staggering acceleration compared to the uncatalyzed reduction of N_2_ under physiological conditions.[^4a^, ^6^]

### 1.2 Nitrogenases: isozymes, structures and mechanism

The enzyme family of nitrogenases consists of the Mo nitrogenase, the most abundant and best studied nitrogenase, and the two alternatively expressed nitrogenases: the vanadium (V) and the iron‐only (Fe‐only) nitrogenase.[^4b^, ^7^] While all known diazotrophic organisms encode the Mo nitrogenase, some diazotrophs harbor one of the alternative nitrogenases and even less organisms encode all three nitrogenases at the same time.^[8]^ Both alternative nitrogenases likely function as fail‐safe enzymes in Mo depleted environments. The V and Fe‐only nitrogenases are thought to have arisen from the Mo nitrogenase and all three nitrogenases share a high degree of sequence identity. For example, the Mo nitrogenase of the model organism *Azotobacter vinelandii* has a sequence identity of 44.0 % and 36.7 % with the V and the Fe‐only nitrogenase, respectively.^[9]^ The corresponding genes are named *nif, vnf* and *anf* for the Mo, V and Fe‐only nitrogenase, respectively. The protein structures of the Mo and V nitrogenase are strikingly similar.^[10]^ Spectroscopic studies and the sequence identity indicate that the structure of the Fe‐only nitrogenase closely resembles the other two nitrogenases.^[11]^

**Figure 1:**
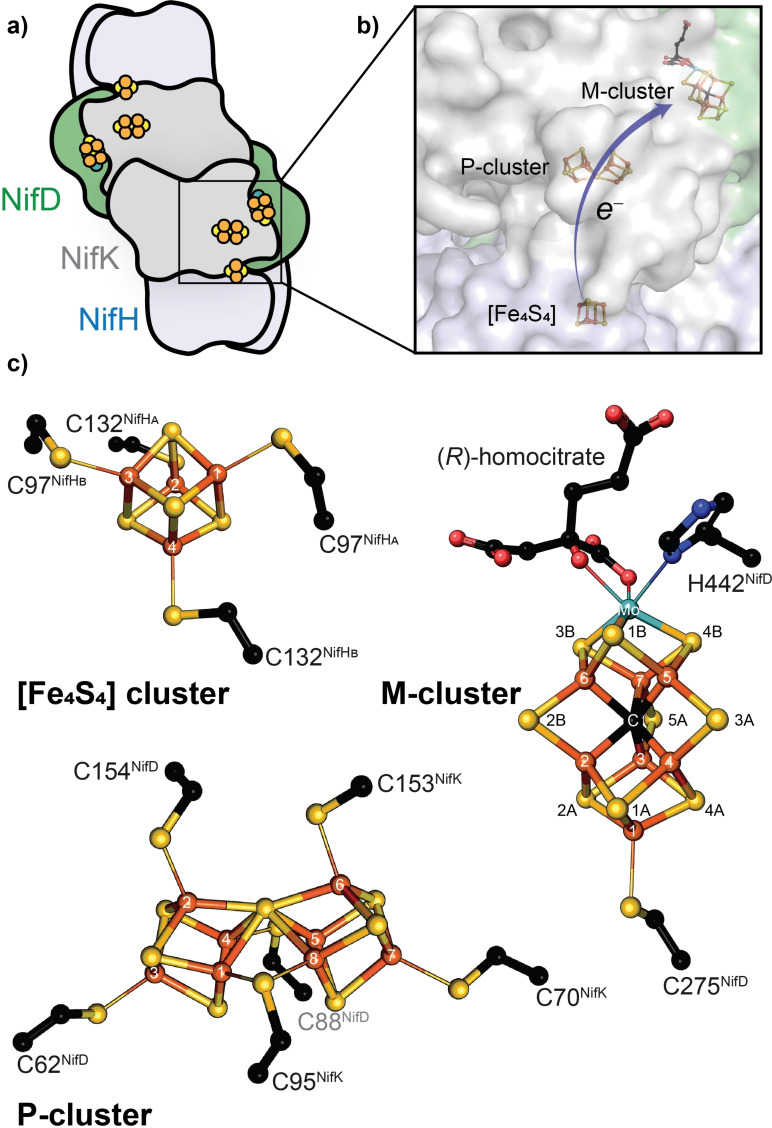
The Mo nitrogenase structure of *A. vinelandii*. a) The catalytic component (Nif(DK)_2_ or MoFe protein) bound to two reductase components (NifH_2_ or Fe protein). b) Close‐up of the metal cluster location inside the Mo nitrogenase (PDB 4WZA). c) Atomic structure of the three nitrogenase metal clusters: [Fe_4_S_4_] cluster, P‐cluster and M‐cluster.

All known nitrogenases consist of a catalytic component and a reductase component. The catalytic component is called MFe protein (MoFe protein, VFe protein, FeFe protein, according to the metal content). The reductase component is termed Fe protein, for all three nitrogenases. The MoFe protein is encoded by *nifDK* and forms a (*αβ*)_2_‐heterotetramer (Figure 1a, 3a). Each *αβ*‐subunit forms an independent catalytic unit harboring two metallic cofactors, the [Fe_8_S_7_] P‐cluster involved in the electron transfer and the M‐cluster (also called FeMoco) in the active site of the Mo nitrogenase (Figure 1c). The analogous catalytic components of the V and the Fe‐only nitrogenase are encoded by *vnfDGK* and *anfDGK*, respectively, forming an (*αβγ*)_2_‐heterohexamer (Figure 3b). This hexamer possesses two P‐clusters and two active site cofactors (V‐cluster and Fe‐cluster, respectively). The function of VnfG and AnfG is unknown, but an involvement in V/Fe‐only nitrogenase maturation as well as Fe protein and VFe/FeFe protein complex formation and electron transfer have been suggested.^[12]^ The Fe proteins encoded by the *nifH*, *vnfH* or *anfH* gene form homodimers with a subunit bridging [Fe_4_S_4_] cluster responsible for electron delivery.

The three different active site cofactors (M‐, V‐, Fe‐cluster) are the most complex metallocofactors observed in nature and constitute the most apparent difference between the three isozymes. The M‐cluster has the chemical composition of [MoFe_7_S_9_C‐(*R*)‐homocitrate]. Six Fe atoms form a trigonal prism around an interstitial μ_6_‐C atom that is capped with three μ_2_‐S^2−^ ligands (S2B, S3A, S5A), the belt sulfides (Figure 1c).^[13]^ The trigonal faces of the prism are both connected to three μ_3_‐S^2−^, one leading to the terminal Fe atom that is linked to the protein scaffold by C275 (numbers according to NifD of *A. vinelandii* are used below, except indicated otherwise) and the opposite side is connected to the octahedrally coordinated Mo atom. The Mo atom is further coordinated by the bidentate ligand *R*‐homocitrate and H442 of NifD. The V‐cluster has the composition [VFe_7_S_8_C(CO_3_)‐(*R*)‐homocitrate] with the S3A belt sulfide being replaced by a CO_3_^−^‐ion, and V in place of Mo (Figure 3c).^[14]^ The Fe‐cluster composition is not verified by X‐ray crystallography, but spectroscopic investigations suggest a composition of [Fe_8_S_9_C‐(*R*)‐homocitrate] with Fe at the Mo position.^[11a]^

In addition to C275 and H442, the M‐cluster is surrounded by highly conserved residues, i. e. V70, R96, Q191 and H195, which participate in the N_2_ reduction mechanism.^[15]^ R96, Q191 and H195 are involved in proton transport and substrate binding.^[16]^ V70 regulates substrate access to the cofactor by steric control.^[17]^

**Figure 2:**
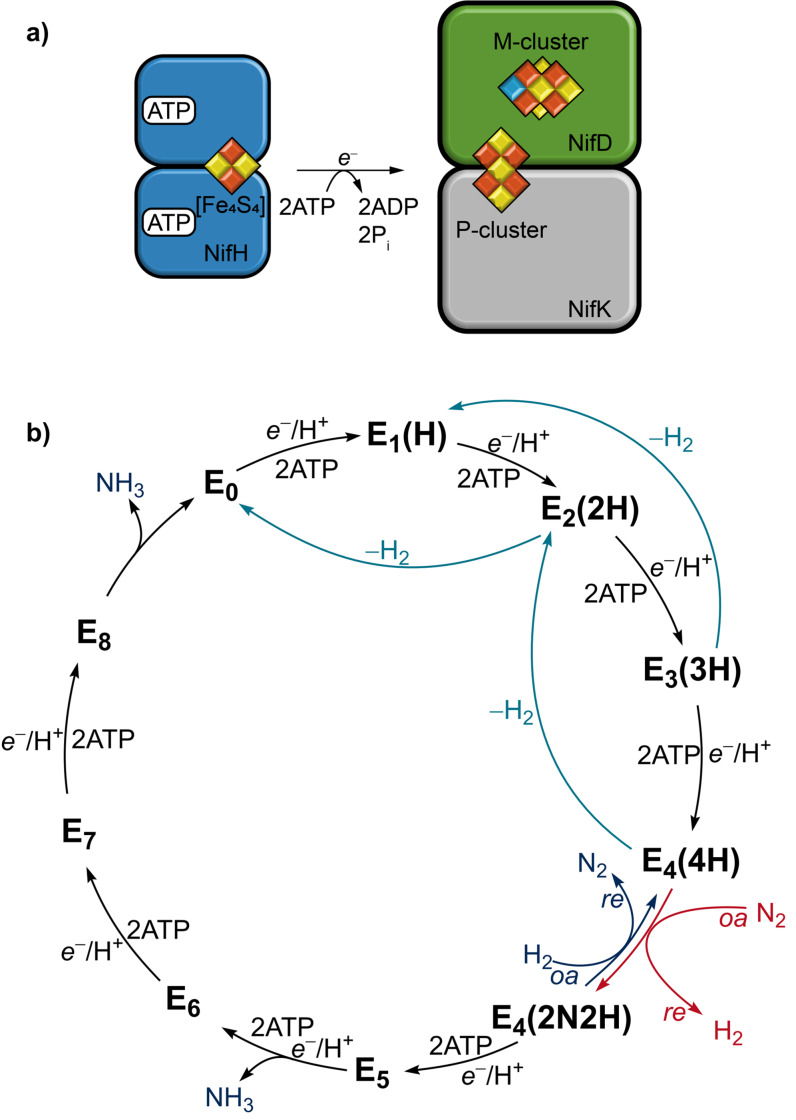
a) ATP‐driven association of the reductase component (NifH_2_ homodimer) to the catalytic component (for clarity, only one NifDK *αβ*‐subunit of the heterotetramer is depicted). b) Simplified version of the Lowe–Thorneley kinetic scheme for nitrogen reduction. For each integer n, an additional *e* ^−^/H^+^ is added to the M‐cluster by the Fe protein and proton channeling. The H and N stoichiometry is given in parentheses.

The overall catalytic mechanism of nitrogenases has been characterized.[^4^, ^18^] However, the exact catalytic conversion of substrates at the M‐cluster is still under debate.[^16a^, ^19^] The two processes vital for nitrogenase activity are the electron flux and the electron accumulation and substrate conversion at the M‐cluster. They are described by the model of ‘deficit‐spending’ and the Lowe–Thorneley reaction scheme, respectively (Figure 2).[^4a^, ^20^]

The electron flow in nitrogenases originates from the Fe protein and proceeds via the P‐cluster to the M‐cluster (Figure 1b, 2a). The Fe protein is an electron donating enzyme that delivers the required electrons for substrate reduction. For electron transfer, the [Fe_4_S_4_] cluster of the Fe protein is first reduced from the [Fe_4_S_4_]^2+^ state to the [Fe_4_S_4_]^1+^ state by ferredoxins or flavodoxins. Next, ATP binds to the Fe protein, triggering a structural rearrangement and allowing the reductase component to bind to the catalytic component, forming the nitrogenase complex. Upon complex formation between the Fe and the MoFe protein, the reduction potential of the P‐cluster is lowered and one electron is transferred to the M‐cluster.^[21]^ This leaves the P‐cluster in its oxidized state P^1+^, which is re‐reduced by the Fe protein. The reduction of the M‐cluster with subsequent re‐reduction of the P^1+^‐cluster is denoted as ‘deficit‐spending’. Each electron transfer is accompanied by the hydrolysis of two molecules of ATP and followed by the dissociation of the Fe protein from the catalytic component.^[22]^ Repeated association and dissociation of the Fe protein results in a constant flow of electrons to the M‐cluster for substrate reduction.

The Lowe–Thorneley scheme has been proposed on the basis of kinetic and spectroscopic investigations of the Mo nitrogenase. According to this scheme, the electron flux subsequently introduces additional electrons to the M‐cluster, starting from the most oxidized **E_0_** state (Figure 2b).^[4a]^ The subscript number counts the added electrons and protons (supplied via proton channeling). After at least two electrons and protons are added to the M‐cluster (**E_2_**, **E_3_**, **E_4_**), the spontaneous release of H_2_ gas can occur, leading from the **E_n_** state to the **E_n‐2_** state (2≤n≤4). After four electrons and protons are added to reach the **E_4_** state, an exchange of the two bound hydrides by N_2_ occurs via a coupled reductive elimination (*re*) of the hydrides under release of H_2_ and oxidative addition (*oa*) of N_2_.^[23]^ From this **E_4_** (**2N2H**) state, subsequent electron flow leads to the full reduction of N_2_ to two equivalents of NH_3_ without the release of H_2_. This renders the **E_4_** state the critical intermediate for N_2_ reduction, called Janus‐Intermediate.^[24]^

The same kinetic scheme can be used for the alternative nitrogenases, though their optimum N_2_ fixation stoichiometry shows an increased ATP consumption and H_2_ production compared to the Mo nitrogenase.[^7b^, ^25^] Nitrogenases can be compared by analyzing their overall electron flux and electron usage (Table 1). For *A. vinelandii* the Mo and V nitrogenase exhibit a conversion of 11–14 *e* ^−^×protein^−1^×s^−1^, under high electron flux (Fe protein is present in large excess)_._ ^[23b]^ While the Mo nitrogenase uses 62 % of the electron flux for N_2_ reduction, only 34 % is used for N_2_ reduction by the V nitrogenase.^[23b]^ The N_2_ reduction activity of the Fe‐only nitrogenase from *A. vinelandii* is further lowered by, both, a nearly halved overall electron flux of 7 *e* ^−^×protein^−1^×s^−1^ and only 31 % of the *e* ^−^ are directed towards N_2_ reduction.^[23b]^ Consistent with the decrease in activity from the Mo over the V to the Fe‐only nitrogenase, is the distribution of nitrogenases in nature. The Mo nitrogenase is the most abundant nitrogenase encoded by all known diazotrophs. Whereas the V and the Fe‐only nitrogenase occur only in 6.4 % and 8.9 % of the sequenced diazotrophs, respectively.^[26]^ Furthermore, the alternative nitrogenases are transcriptionally repressed if Mo is present in the environment.^[27]^ Differences in activity, specificity and product profile are commonly observed for the three nitrogenase isozymes. In the case of N_2_ reduction, differences between the Mo, V and Fe‐only nitrogenase can likely be attributed to the different active site cofactors. The terminal transition metals (Mo, V, Fe) in the active site cofactor mainly contribute to the spin‐state fine‐tuning.^[28]^ It is hypothesized that the terminal transition metals are not directly involved in the substrate binding.

**Table 1:** Comparison of the *A. vinelandii* nitrogenases’ electron flux towards N_2_ reduction, H_2_ production and the total electron flux usage under a pure nitrogen atmosphere.^[a]^

Protein	N_2_	H_2_	Total	N_2_ flux [%]	MFe : Fe^[b]^
MoFe	7.26	4.38	11.64	62	20 : 1
VFe	4.80	9.06	13.86	34	20 : 1
FeFe	2.25	4.97	7.22	31	30 : 1

[a] Data for 1 atm nitrogen pressure, from Ref. [23b]. Electron fluxes are reported as *e* ^−1^×protein^−1^×s^−1^. [b] Ratios of MFe protein to Fe protein, all values correspond to high electron flux conditions.

Although the described differences in electron flux and product profiles originate from assays with *A. vinelandii* nitrogenases, each subset of nitrogenases from other organisms reacts generally in similar ways.^[4b]^ Similar activity patterns of the Mo, V and Fe‐only nitrogenase from different organisms indicate that the main difference among these proteins arises from the active site cofactor rather than the highly conserved protein environment.

### 1.3 Substrate range of nitrogenases

For the conversion of N_2_, the M‐cluster accumulates electrons and protons until the Janus‐Intermediate **E_4_** is reached. In this process, each added electron is either stored in the reduced state of the M‐cluster (**E_1_**, **E_3_**) or pairwise in hydrides bound to the M‐cluster (**E_2_**, **E_4_**).[^18^, ^29^] The hydrides on the active site cofactor enable nitrogenases to reduce additional small compounds. In fact, nitrogenases have one of the broadest substrate scopes known for enzymes. Besides their natural substrates H^+^ and N_2_, nitrogenases can reduce non‐physiological nitrogen compounds (azide [N_3_^−^],^[30]^ nitrite [NO_2_^−^]^[31]^), carbon–nitrogen compounds (cyanide [CN^−^]^[32]^), (iso)nitriles (HC≡N, RC≡N, RN≡C),^[33]^ unsaturated hydrocarbon compounds (acetylene [C_2_H_2_],^[34]^ cyclopropene^[35]^), and carbon–chalcogen compounds (carbon monoxide [CO],^[36]^ carbon dioxide [CO_2_])^[37]^ (see Table 2). This incomplete enumeration illustrates the high reactivity of nitrogenases. The full substrate scope of nitrogenases has recently been reviewed elsewhere.[^4b^, ^4c^, ^38^]

**Table 2:** Selection of substrates reduced by nitrogenases.

Substrates	Reaction					Ref.
**Nitrogen substrates**						
Azide	HN_3_+2H^+^+2*e* ^−^→N_2_+NH_3_					[30]
Nitrite	NO_2_^−^+7H^+^+6*e* ^−^→NH_3_+2H_2_O					[31]
	
**Carbon‐nitrogen substrates**						
Cyanide	HC≡N+6H^+^+6*e* ^−^→CH_4_+NH_3_					[32]
Nitriles	RC≡N+6H^+^+6*e* ^−^→RCH_3_+NH_3_					[33a,b]
Isonitriles	RN≡C+6H^+^+6*e* ^−^→RNH_2_+NH_3_					[33c]
	
**Carbon‐carbon substrates**						
Acetylene	HC≡CH+2H^+^+2*e* ^−^→H_2_C=CH_2_					[34]
Cyclopropene	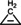	+2H^+^+2*e* ^−^→	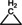	or	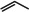	[35]
	
**Carbon‐chalcogen substrates**						
Carbon monoxide	C≡O+6H^+^+6*e* ^−^→CH_4_+H_2_O					[36]
Carbon dioxide	O=C=O+8H^+^+8*e* ^−^→CH_4_+2H_2_O					[37]

Most of the unnatural substrates were studied to investigate the mechanism of nitrogenases. CO has been extensively used to investigate the nitrogenase cofactors beyond the resting state **E_0_**. The properties of CO allowed the trapping of CO bound intermediates under turnover conditions and their characterization by infrared (IR) and electron paramagnetic resonance (EPR) spectroscopy as well as X‐ray crystallography. These investigations led to the elucidation of the substrate binding by the nitrogenase active site cofactor. Nonetheless, the ‘side reactivities’ of nitrogenases potentially have physiological relevance, e. g. the conversion of cyanide and CO was speculated to contribute to cellular detoxification.^[39]^ Furthermore, the reduction of CO_2_ to methane (CH_4_) by nitrogenases has spiked interest for its potential role in shaping microbial communities.^[9b]^ The direct reduction of CO_2_ is particularly noteworthy, as it places nitrogenases in a small group of enzymes with the same ability, i. e. nickel‐iron CO dehydrogenases and formate (HCOO^−^) dehydrogenases, which catalyze the reversible reduction of CO_2_ to CO and HCOO^−^, respectively.^[40]^

Beyond the biological importance of these reactivities, the conversion of CO_2_ into hydrocarbons suggests an industrial use of nitrogenases for carbon capture and the production of biofuels in a green economy. Due to the increasing importance of carbon capture and biofuel production, we review and discuss the current knowledge of the CO and CO_2_ conversion by all three nitrogenase isozymes: the Mo, V and Fe‐only nitrogenase.

## 2 CO Reduction by Nitrogenases

Carbon monoxide is a colorless toxic gas that is isoelectronic to N_2_. In contrast to N_2_, CO is a strong *π*‐acid due to its low energy π*‐orbital. This allows CO to easily bind to transition metals by synergistic *σ* donation/*π* backdonation between the transition metal d‐orbitals and the frontier molecular orbitals of CO. Thus, CO is one of the broadly used ligands in transition metal chemistry and likewise strongly interacts with the metal cofactors of the nitrogenases. CO has further been used as a C_1_ building block for the synthesis of hydrocarbons in the Fischer–Tropsch process.^[41]^ In this process, syngas (a mixture of CO and H_2_) is produced by gasification of coal or steam reforming and passed over heterogeneous iron or cobalt catalysts at 300–350 °C to produce hydrocarbons. The nitrogenase catalyzed conversion of CO and H^+^ to hydrocarbons could be a green alternative to the Fischer–Tropsch process. Consequently, nitrogenases parallel two important industrial processes: the Haber–Bosch process for fertilizer production and the Fischer–Tropsch process for fuel production.

### 2.1 CO reduction by the Mo nitrogenase

Early studies showed that CO is a potent noncompetitive inhibitor for the reduction of N_2_, N_3_^−^, C_2_H_2_, and CN^−^ catalyzed by the Mo nitrogenase.^[42]^ While low partial pressures of CO (pCO=0.005 atm) fully inhibit the reduction of N_2_, the hydrolysis of ATP and the formation of H_2_ remain unaffected in the presence of CO. EPR analysis of the Mo nitrogenase under turnover conditions in the presence of CO revealed two new EPR signals.^[43]^ One signal was observed under low CO partial pressures ([CO]=[MoFe protein]) and was denoted lo‐CO (*S*=1/2). The second signal, formed at higher CO concentrations, was denoted hi‐CO (*S*=1/2). Labeling the MoFe protein with ^57^Fe led to a signal broadening by nuclear hyperfine interactions, demonstrating that the EPR signal arose from one of the Fe‐containing clusters of the MoFe protein. Both, the uninhibited H_2_ production of the wild type (WT) Mo nitrogenase and the appearance of distinct EPR signals assigned to the CO‐bound MoFe protein renders the CO inhibition assays a powerful tool for the investigation of nitrogenases.

Following this strategy, Lowe *et al*. investigated the C_2_H_2_ reduction mechanism of the Mo nitrogenase under CO. Under high electron flux the formation of H_2_ was inhibited by C_2_H_2_. The addition of CO together with C_2_H_2_ to the headspace did not restore the uninhibited H_2_ formation rate. The authors therefore suggested that the Mo nitrogenase has different binding sites for CO and C_2_H_2_.^[44]^ The pH range of the Mo nitrogenases is determined by two groups at p*K*_a_ values of 6.3 and 9.0.^[45]^ Turnover under CO shifts the basic p*K*_a_ from 9.0 to 8.5, leading to an apparent inhibition of H_2_ formation under basic conditions (pH>8.5). Newton and co‐workers studied different NifD mutations (H195N, Q191K, H195Q, R96Q, R96K, R359K, R277C, R277H, R96K/R359K) to identify the M‐cluster position in the protein and to illuminate important residues for nitrogenase catalysis.[^15^, ^46^] The effects of the mutations on the M‐cluster environment were probed by the CO inhibition of the H_2_ formation and changes in the lo‐CO and hi‐CO EPR signals. Comparing the H_2_‐formation under argon, all mutants exhibited a decreased electron flow relative to the WT MoFe protein. The H_2_ formation of the mutants R96K, Q191K, R359K and R96K/R359K was additionally inhibited by CO. Except for R96Q, none of the investigated mutants exhibited the hi‐CO state.

Further spectroscopic studies were conducted to better characterize the lo‐CO and hi‐CO states. ^13^C electron nuclear double resonance (ENDOR) spectroscopy revealed the binding of one or two CO molecules in the lo‐CO or hi‐CO signal, respectively, at the same metal cluster of the MoFe protein.^[47]^ Both species could also be interconverted by pumping off or adding CO without the occurrence of a redox step. This suggests that the lo‐ and hi‐CO signal originate from the same **E_n_** state of the MoFe protein. Selectively labeling the M‐cluster with ^57^Fe confirmed the M‐cluster as the CO binding site and excluded the possible binding of CO to the P‐cluster.^[48]^ The hyperfine tensors of the ^13^CO‐labeled lo‐ and hi‐CO states, derived from orientation‐selective ^13^C ENDOR spectroscopy, located the binding site of CO in the belt region of the M‐cluster. An Fe bridging μ‐CO was identified for the lo‐CO and two terminally bound CO molecules were observed for the hi‐CO state.^[49]^ A new MoFe protein species with two bound CO molecules was observed under high electron flux by EPR spectroscopy (*S*=3/2), known as hi(5)‐CO.^[50]^ While the lo‐CO and hi(5)‐CO species are photostable, hi‐CO can be converted to lo‐CO by light irradiation, which suggests structural differences between the hi‐CO and hi(5)‐CO species.^[51]^ Moreover, CO causes a strong characteristic signal in IR‐spectra, independent of the cofactor's spin state. Thus, CO was extensively used as an IR‐probe to investigate the nitrogenase inhibition and the cofactor environment.[^17^, ^52^] Stopped‐flow FT‐IR and IR‐monitored photolysis implied a more complex ensemble of different species in samples prepared under hi‐CO conditions.[^52a^, ^52c^] In the IR‐monitored photolysis experiments, a species with difference bands at 1936 cm^−1^ and 1858 cm^−1^ was observed, which likely corresponds to the hi‐CO, *S*=1/2 EPR species.^[52a]^ A second species with difference bands at 1936 cm^−1^ and 1858 cm^−1^ was observed in stored samples. The second species was assumed to correspond to the hi(5)‐CO species. The two species were denoted Hi‐1 and Hi‐2. Some of the CO‐bound species were investigated via nuclear resonance vibrational spectroscopy (NRVS) and extended X‐ray absorption fine structure (EXAFS) spectroscopy.^[53]^ Both methods indicated a symmetry reduction in the central Fe_6_S_9_C core of the M‐cluster upon CO binding. Taken together the spectroscopic studies with density functional theory (DFT) calculations, the Fe‐2,3,6,7 face of the M‐cluster was identified as a potential binding site for CO and a structure with a μ‐CO between Fe(2) and Fe(6) was proposed.

The CO binding was further characterized, by driving the MoFe protein electrochemically using Eu(II)L/Eu(III)L (L=polyaminocarboxylate) complexes as direct electron mediators to replace the Fe protein.^[54]^ Comparing the potentials required for H_2_ formation and CO binding to the MoFe protein, which both coincide close to −900 mV, it was suggested that CO binding occurs in a state generated during the catalytic H^+^ reduction cycle of the MoFe protein. With regard to the Lowe–Thorneley scheme, this state could be **E_2_**, as previously proposed.^[55]^

**Figure 3:**
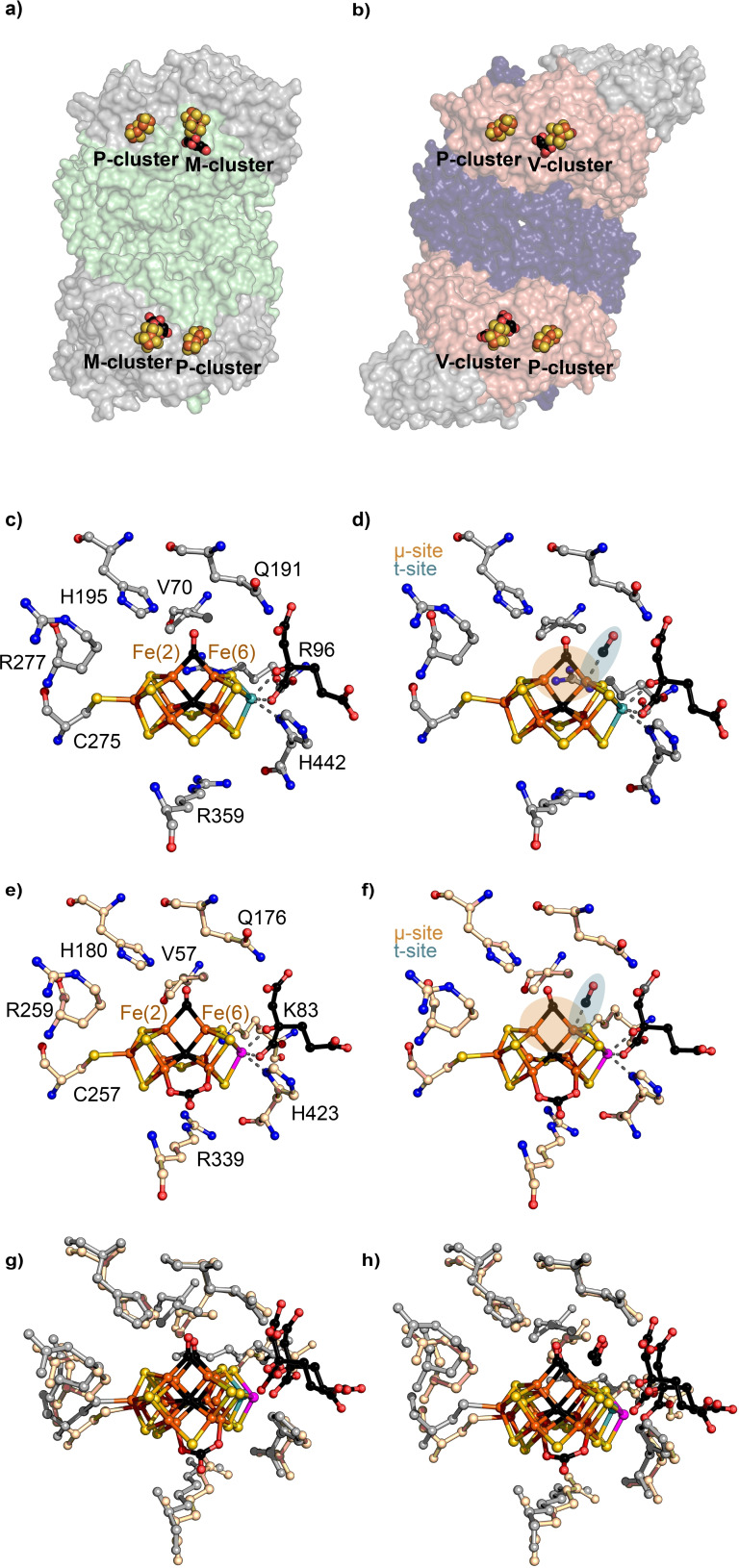
Structures of the Mo and V nitrogenase catalytic components and the M‐ and V‐clusters upon CO incubation. a) Structure of Nif(DK)_2_ heterotetramer (NifD, grey; NifK, green; PDB 4WZA) b) structure of the Vnf(DGK)_2_ heterohexamer (VnfD, red; VnfK, violet; VnfG, grey; PDB 5 N6Y) c) CO‐bound active site of the MoFe protein in the lo‐CO state (PDB 4TKV) and d) hi‐CO state (PDB 7JRF). e) CO‐bound active site of the VFe protein in the lo‐CO state (PDB 7ADR) and f) hi‐CO state (PDB 7AIZ). g) Overlay of the lo‐CO structures of the M‐[c)] and V‐cluster [e)] and h) overlay of the hi‐CO structures of the M‐[d)] and V‐cluster [f)].

Finally, the CO‐inhibited MoFe protein structure reported by Spatzal *et al*. provided new insights into the binding mode of CO to the M‐cluster (Figure 3c).^[56]^ The CO‐bound MoFe protein was isolated from a CO activity assay and subsequently crystallized. The M‐cluster contained a bridging μ‐CO ligand, which replaced the S2B belt sulfur between the Fe(2) and Fe(6) atoms. Thus, transfer of electrons and protons proceed the reversible replacement of the S2B sulfide. A potential sulfur storage site, binding the sulfur during turnover, was identified. Both findings imply that the activation of the M‐cluster via the exposure of the Fe(2) and Fe(6) face is also a crucial step in the reduction of N_2_. More evidence for this hypothesis is added by the fact that CO binds to the M‐cluster only under turnover conditions and cannot interact with the cofactor in the **E_0_** resting state, although the lo‐CO state has the same oxidation level as **E_0_**. This indicates that the Fe(2)–Fe(6) remains protected and inaccessible for CO in the **E_0_** resting state. Moreover, the first CO‐bound structure of the Mo nitrogenase closely resembles the proposed structure of the lo‐CO state.^[49]^ Crystals of the Mo nitrogenases in the hi‐CO state were obtained from lo‐CO single crystals by placing the lo‐CO crystals under CO overpressure.^[57]^ The structure of hi‐CO showed an additional CO molecule terminally bound to the Fe(6) atom with an occupancy of q=0.5 (t‐site, Figure 3d), but otherwise remained nearly identical compared to the lo‐CO structure. The CO‐bound structures represent the lo‐CO and hi‐CO states as confirmed by EPR spectroscopy.^[57]^

After discovering that the V nitrogenase reduces and couples CO to C_1≤4_ hydrocarbons, the activity of the Mo nitrogenase towards CO reduction was revisited by Ribbe and co‐workers.[^36a^, ^58^] In agreement with the lack of H^+^ reduction inhibition by CO in the Mo nitrogenase, the total specific activity for hydrocarbon formation of the Mo nitrogenase is rather low, only ∼0.1 % of the activity observed for the V nitrogenase. The hydrocarbons produced by the Mo nitrogenase include C_2_H_4_, C_2_H_6_, C_3_H_6_ and C_3_H_8_, but CH_4_ was not detected. The CO reduction behavior is further influenced by replacing H_2_O with D_2_O in the buffer. In addition to the previously observed hydrocarbon products, D_2_O stimulated the formation of the longer hydrocarbon chains *α*‐C_4_D_8_ and *n*‐C_4_D_10_. Besides optimizing the buffer conditions, mutants with improved CO reduction activity were identified. The V70A mutation in NifD increases the space surrounding Fe(2)–Fe(6) CO‐binding site and results in an increased hydrocarbon formation activity (Figure 3).^[36b]^ Changes in CO partial pressure and electron flow as well as additional mutations in the cofactor environment further modulated the CO reduction activity and product distribution.

The formation of hydrocarbons triggered the question whether the interstitial carbon of the M‐cluster is involved in the conversion of CO. However, the interstitial carbon of the M‐cluster is likely not interchangeable, as ^13^C‐labeling of the M‐cluster did not result in the release of ^13^C‐containing hydrocarbons under turnover conditions.^[59]^

To summarize this chapter, the interaction of CO with the M‐cluster has been known since the 1970s and was characterized by mutational and spectroscopic means. CO was further used as a tool for the investigation of the Mo nitrogenase structure and mechanism. The validity and importance of these CO studies were highlighted by the discovery that the Mo nitrogenase can reduce CO to hydrocarbons. The capability of the Mo nitrogenase to bind and reduce CO suggests that the structurally elucidated sites of CO binding are the general sites of substrate binding in the M‐cluster.

### 2.2 CO reduction by the V nitrogenase

Under CO, the H_2_ formation of the V nitrogenase is inhibited by an average of 35 % without a decrease in the ATP consumption rate.^[36a]^ The missing electron flow is redirected into the reduction of CO to hydrocarbons in a Fischer–Tropsch‐like reaction, as demonstrated by Ribbe and co‐workers.[^36a^, ^58^] The formed hydrocarbons are CH_4_, C_2_H_4_, C_2_H_6_, C_3_H_6_, C_3_H_8_, *α*‐C_4_H_8_ and *n*‐C_4_H_10_. C_2_H_4_ is the main product, accounting for 94 % of the total hydrocarbon yield.^[58]^ The activity of the V nitrogenase corresponds to an 800‐fold increase in hydrocarbon formation compared to the Mo nitrogenase. The hydrogen source of the produced hydrocarbons was traced to H^+^ that originated from the buffer.^[60]^ While the conversion of CO by the V nitrogenase in D_2_O led to the formation of deuterated hydrocarbons, no incorporation of deuterium into the products was detectable for turnover under D_2_ gas. In contrast to the Mo nitrogenase, changing the buffer from H to D only slightly effected the CO reduction and the hydrocarbon product distribution of the V nitrogenase. The different impact of D on the Mo and V nitrogenase catalysis suggests differences in the coupled electron/proton tunnelling of this reaction.^[58]^

Due to the CO reduction rates by the V nitrogenase, the isolation of the V nitrogenase corresponding to lo‐CO and hi‐CO states is hampered under turnover conditions, *i. e*. under electron influx through the reductase component. However, an intermediate of the VFe protein binding one CO molecule was obtained by using the strong reductant [Eu(II)‐DTPA] as electron source.^[61]^ This intermediate exhibited a similar EPR spectra as the lo‐CO state of the Mo nitrogenase. The reversibility of the CO binding was demonstrated by releasing the bound CO. Putting the lo‐CO VFe protein under turnover conditions (adding VnfH and ditionite) resulted in the release of CH_4_. The catalytic relevance of the isolated lo‐CO state was further demonstrated by turning over ^13^CO labeled lo‐CO VFe protein in the presence of ^12^CO, to release the partially labeled ^13^CH_2_^12^CH_2_ product.

For the first time, the hi‐CO EPR signal of VnfDGK was observed after reducing CO under a decreased electron flux, by exchanging the VnfH of *A. vinelandii* with the VnfH of *Methanosarcina acetivorans*.^[62]^ The hi‐CO state of VnfDGK was later also isolated by exposing VnfDGK to an increased CO pressure and using [Eu(II)‐DTPA] as reductant.^[63]^ The second CO molecule bound to the V‐cluster was not part of the C−C coupling reaction and it was suggested that the second CO binding site is independent from the CO conversion to hydrocarbons.

Recently, the X‐ray crystal structures of the lo‐CO and hi‐CO states of the V nitrogenase have been reported by Einsle and co‐workers. These structures gave further insights into the VFe active site (Figure 3e, f).^[64]^ The lo‐CO and hi‐CO structures of the VFe protein closely resemble the homologous structures of the MoFe protein. All structures exhibit a μ‐CO bridging Fe(2) and Fe(6) and a terminal bound CO in the hi‐CO state (Figure 3e, h). The residues of the MO nitrogenase shown to affect the lo‐CO state upon mutation, exhibit similar conformations in the structures of the CO bound MoFe and VFe protein. However, residue R96 of NifD is replaced by lysine (K83) in VnfD and as described above, one of the belt sulfides in the V‐cluster is replaced by a CO_3_^−^‐ligand (Figure 3c). How these changes can account for the large difference in Mo and V nitrogenase activity is not obvious. The origin of the reactivity difference is likely to be more subtle. Single crystals of the VFe protein in the lo‐CO state could partially be interconverted to the hi‐CO state under CO overpressure, with the occupancy of q=0.5, as observed for the Mo nitrogenase. The binding and dissociation process of the terminally bound CO was completely reversible within 100 s, as monitored by *in situ* attenuated total reflectance IR difference spectroscopy.

Beyond the *in vitro* characterization of the CO binding and activation by nitrogenases, it was demonstrated in the model organism *A. vinelandii* that the V nitrogenase reduces CO to hydrocarbons also *in vivo*.^[65]^ After expression of the V nitrogenase, the cultures were incubated under 15 % CO for 8 h. C_2_H_4_, C_2_H_6_ and, C_3_H_8_ were formed and the turnover number was determined to be ∼750. The hydrocarbon yield could be further increased by incubating the cultures alternately under an air and a 15 % CO atmosphere. Alternating the environmental gas composition allowed *A. vinelandii* to recover from phases with CO gas. The achieved specific activity for the *in vivo* C_2_H_4_ formation (1 mmol C_2_H_4_×[g dry cell]^−1^×h^−1^) was already in the range of other bio‐ethylene formation methods without any optimization.

The CO‐reducing and C−C coupling activity of the V nitrogenase renders this enzyme system as a potential biological alternative to the industrial Fischer–Tropsch process for fuel formation.

### 2.3 CO reduction by the Fe‐only nitrogenase

The H_2_ formation catalyzed by the Fe‐only nitrogenase is progressively inhibited by increasing CO concentrations until it reaches an inhibition of 35 % at a CO partial pressure of 0.4 atm.^[66]^ In contrast to the V nitrogenase, the Fe‐only nitrogenase reduces CO mainly to CH_4_ with a maximum specific activity at a CO partial pressure of ∼0.05 atm. The CH_4_ formation activity of the Fe‐only nitrogenase decreased to 10 % of the maximum value, when the CO partial pressure was further increased. Therefore, the CH_4_ formation alone cannot fully account for the observed inhibition of H_2_ formation at elevated CO concentrations and either CO inhibits the electron flux of the Fe‐only nitrogenase or an undetected product is formed.^[66]^

The differences in the product profile between the V nitrogenase (mainly C_2_H_4_) and the Fe‐only nitrogenase (only CH_4_) is so far unexplained, as no structural or spectroscopic characterization of the Fe‐only nitrogenase under CO has been carried out. However, a similar product shift was observed for the CO reduction by the isolated V‐ and L‐cluster under [Eu(II)‐DTPA].^[67]^ The L‐cluster is the cofactor precursor of the M‐cluster with the chemical composition [Fe_9_S_9_C]. The L‐cluster closely resembles the proposed structure of the Fe‐cluster except that (*R*)‐homocitrate is missing. The preferred reduction of CO to CH_4_ by the L‐cluster indicates that the preference for CH_4_ formation of the Fe‐only nitrogenase could originate from the intrinsic cofactor properties rather than the protein environment. Further investigations are needed to elucidate the CO conversion by the Fe‐only nitrogenase.

### 2.4 Comparison of the Mo, V and Fe‐only nitrogenase

The specific activity for CO reduction is increasing from the Mo nitrogenase (traces) through the Fe‐only nitrogenase (4.5 nmol CH_4_×[nmol FeFe protein]^−1^×min^−1^) to the V nitrogenase (7.5 nmol C_2_H_4_×[nmol FeFe protein]^−1^×min^−1^).^[66]^ The pronounced disparities in the CO reduction activity between the Mo and V nitrogenase raise the question whether these disparities originate from the different protein environments or the different cofactor compositions.

The isolated M‐cluster and V‐cluster are able to produce hydrocarbons from CO at similar rates by using the strong reductants [Eu(II)‐DTPA] or SmI_2_ as electron sources.[^67^, ^68^] Still, the rate of the isolated V‐cluster only corresponds to 0.05 % of the activity reached by the V nitrogenase, highlighting the strong influence of the V nitrogenase protein. This was further confirmed by investigating the CO‐reduction behavior of a V‐cluster containing MoFe protein (NifDK^V^) and an M‐cluster containing VFe protein (VnfDGK^M^).^[69]^ The hydrocarbon formation activity of NifDK^V^ was 640 times smaller as the one observed for VnfDGK.^[69a]^ Similarly, the hydrocarbon formation activity for VnfDGK^M^ increased by 100‐fold compared to NifDK.^[69b]^ Nevertheless, the cofactor also has a significant influence on the CO reduction activity, as VnfDGK was found to be ∼6‐fold more active than VnfDGK^M^.

**Scheme 1:**
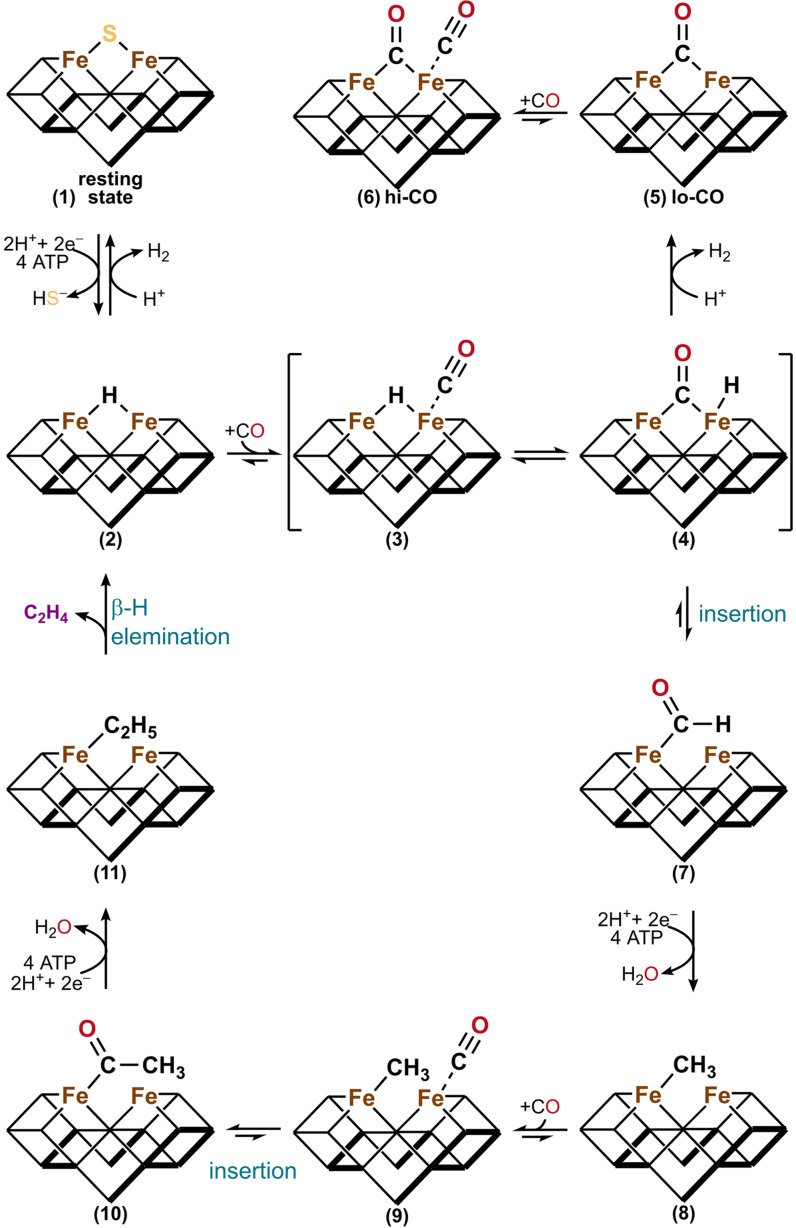
Proposed mechanism of the CO reduction by nitrogenases.^[64b]^ Upon electron flux, the resting state of the cofactor (**1**) acquires two e^−^ and two H^+^ to form the hydride bound **E_2_** state (**2**). CO then binds to the t‐site resulting in adduct (**3**), which undergoes a rearrangement of CO and the hydride to (**4**). The lo‐CO state (**5**) of the cofactor in the formal **E_0_** redox state is then formed by protonation of the hydride in (**4**). The lo‐CO state (**5**) is subsequently interconverted to the hi‐CO state (**6**) under CO overpressure. The intermediate (**4**) alternatively undergoes a CO insertion reaction to the formyl adduct (**7**), which is the starting point of the CO reduction cascade (**8**)–(**11**). After the addition of four extra e^−^/H^+^ and a CO molecule, the ethyl adduct (**11**) is formed. Next, intermediate (**11**) undergoes a β‐H elimination reaction to form the hydride bound **E_2_** state (**2**) and releases C_2_H_4_.

Despite the differences in reactivity, the Mo and V nitrogenase show similar interactions with CO, forming a lo‐ and hi‐CO state, and for both nitrogenases the states are interconvertible. The same behavior and mechanism seem to apply to the Fe‐only nitrogenase. On the basis of the lo‐ and hi‐CO structures of the V nitrogenase, a mechanistic scheme was proposed (Scheme 1).^[64b]^

After reaching the **E_2_** state, CO binds to the t‐site and replaces the hydride at the μ‐site ((**2**)–(**4**)). The reaction can then proceed either by CO insertion of the hydride in (**4**) leading to the formyl adduct (**7**) followed by the reduction of CO to hydrocarbons in a cascade of reductions and a CO addition ((**8**)–(**11**)) or by hydride protonation forming the lo‐CO state (**5**). The lo‐CO state (**5**) can bind an additional CO molecule to form the hi‐CO state (**6**). This mechanism explains why the hi‐CO state cannot couple both bound CO molecules, as the terminal CO at the t‐site of (**6**) has to be exchanged with a hydride to form intermediate (**4**) before the CO reduction to hydrocarbons is initiated. The preference of C_2_H_4_ formation by the V nitrogenase is explained by the preferential β‐H elimination in (**11**) over the methyl protonation in intermediate (**8**). On the other hand, the Fe‐only nitrogenase produces mainly CH_4_, which could be explained by a facilitated protolysis of intermediate (**8**) or by the occurrence of a σ‐bond metathesis between intermediate (**8**) and a generated H_2_ molecule.

To summarize, the activation of CO by the Mo and V nitrogenases seems to depend on the same CO binding mode to the M‐ and V‐cluster. Nonetheless, the CO activity and product profile between the three nitrogenase isoenzymes differs significantly. The activity differences appear to mainly originate from the different protein scaffolds (NifDK, VnfDGK, AnfDGK) as the introduction of the M‐cluster into the V nitrogenase and the reverse exchange, the introduction of the V‐cluster into the Mo nitrogenase, suggest. However, the exact influence of the protein scaffold on the activity could not be clarified, yet.

## 3 CO_2_ Reduction by Nitrogenases

The greenhouse gas CO_2_ is the main source of carbon in the atmosphere. The anthropogenic CO_2_ emissions of the last century increased the abundance of CO_2_ in the earth's atmosphere by 40 %, compared to the pre‐industrial era.^[70]^ The increased CO_2_ concentration is the main contributor to global warming.^[71]^ Besides having a detrimental impact on the environment, the ubiquity of CO_2_ renders it a cheap and accessible source of C_1_ building blocks. Hence, carbon capture to reduce the CO_2_ concentration and conversion of CO_2_ into value‐added products are pressing challenges for chemistry and biology. Recently, nitrogenase proteins were discovered to be capable of activating and reducing CO_2_ to hydrocarbons. Potentially, this reactivity offers new solutions to the great challenge of carbon capture and utilization.

### 3.1 CO_2_ reduction by the Mo nitrogenase

Seelfeldt *et al*. first reported the reduction of CO_2_ to CO by the Mo nitrogenase.^[37a]^ Hemoglobin was used as a CO probe and the formation of CO was monitored by spectrophotometric quantifications of carboxyhemoglobin. This method further prevented the formed CO from inhibiting the Mo nitrogenase during the *in vitro* assay, but was not suitable for the detection of alternative CO_2_ reduction products. The formation of H_2_ during Mo nitrogenase turnover at elevated CO_2_ concentrations (0.45 atm) is inhibited by 16 %.^[72]^ The electrons are redirected towards CO_2_ reduction. HCOO^−^ was later identified by ^1^H and ^13^C NMR spectroscopy as the main product of the CO_2_ reduction by the WT Mo nitrogenase and its mutants. HCOO^−^ is formed at a rate of 9.8 nmol×[nmol MoFe protein]^−1^×min^−1^. The formation of CO is much slower and proceeds at a rate of 0.03 nmol×[nmol MoFe protein]^−1^×min^−1^, although it was discovered first. This product profile was corroborated by quantum mechanical calculations. The reaction pathway of direct hydride transfer from the **E_2_** state to CO_2_, releasing HCOO^−^, is favored over the alternative pathway of CO_2_ activation at the M‐cluster with the reduction of the Fe‐CO_2_ intermediate leading to CO. The product profile of the CO_2_ reduction by the Mo nitrogenase could be extended to CH_4_ by introducing two mutations to the NifD subunit (V70A and H195Q).^[73]^ The formation of CH_4_ likely proceeds over an intermediate CO in a non‐dissociative mechanism. The addition of the CO binder deoxyhemoglobin reduced the amount of formed CH_4_ by 25 % in the CO_2_ reduction assays. The coupling of two or more CO_2_ molecules to longer hydrocarbon chains was not detected. Instead, the Mo nitrogenase could couple C_2_H_2_ and CO_2_ to C_3_H_6_.

Moreover, the CO_2_ reduction behavior of the MoFe protein was electrochemically investigated by immobilizing the MoFe protein in a polymer film on an electrode and cobaltocene was used as the electron mediator.^[74]^ The electrochemical and natural Fe protein‐driven CO_2_ reduction by the MoFe protein showed similar product distributions with 8 % and 9 % of electron flux directed towards the formation of HCOO^−^, respectively. The CO_2_ reduction activity of the Mo nitrogenase double mutant (V75A and H201Q) was further demonstrated *in vivo* using the anoxygenic phototroph *Rhodopseudomonas palustris*.^[75]^ The *in vivo* formation of CH_4_ under photochemotrophic growth is dependent on light for the ATP generation and requires an electron source like acetate (H_3_CCOO^−^) or thiosulfate (S_2_O_3_^2−^). The yield of CH_4_ was further increased by directing metabolic electrons to the nitrogenase proteins. This was partially achieved, either, by preventing further bacterial growth via resuspension of the cells in a nitrogen‐depleted medium or by deletion of the Calvin‐Benson‐Bassham (CBB) cycle from the organism, which is an alternative electron accepting pathway for anoxygenic phototrophs.^[76]^

In short, the Mo nitrogenase converts CO_2_ mainly to HCOO^−^ and a little bit of CO. Nonetheless, Mo nitrogenase mutants can also reduce CO_2_ via the intermediate CO to CH_4_.

### 3.2 CO_2_ reduction by the V nitrogenase

The V nitrogenase can reduce CO_2_ to CO at similar rates as the Mo nitrogenase.^[37b]^ Furthermore, Rebelein *et al*. detected the formation of CD_4_ and the C−C coupling products C_2_D_4_ and C_2_D_6_ in sub‐stoichiometric amounts by changing the medium from H_2_O to D_2_O. In contrast to the V nitrogenase, no C−C coupling products were observed for the Mo nitrogenase. The carbon source for the formed hydrocarbons was traced to CO_2_ by ^13^C‐labeling. Moreover, it was shown that the formation of C_2_D_4_ and C_2_D_6_ proceeds independently from the produced CO, while CD_4_ partially originated from CO. The product distribution of the CO_2_ reduction reaction is modulated, when the reductant [Eu(II)‐DTPA] is used as the electron source.^[77]^ In this [Eu(II)‐DTPA] system, the formation of CH_4_ and the C−C coupling products occur in H_2_O. Furthermore, the product profile is extended from C_2_H_4_ and C_2_H_6_ to C_3_H_6_, C_3_H_8_, C_4_H_8_ and C_4_H_10_. The hydrocarbon formation activity increases dramatically in D_2_O for the V nitrogenase under [Eu(II)‐DTPA] and CO_2_, with the exception of CD_4_, which was not detected under these conditions. Both findings, the longer hydrocarbon chains and the increased product formation indicate that D_2_O facilitates C−C coupling by the V nitrogenase. The ability of the VFe protein to C−C couple CO_2_ to C_2_H_4_ and C_3_H_6_ was also demonstrated in the electrochemically driven CO_2_ reduction by the VFe protein, using 1,1’‐dicarboxycobaltocenium as the electron mediator.^[78]^ Moreover, the V nitrogenase forms detectable amounts of CH_4_ from H_2_CO_3_ inside *R. palustris*. Increased amounts of CH_4_ were observed in strains expressing a double mutant of the VFe protein (V57A and H180Q in VnfD).^[8]^

To summarize, also the V‐nitrogenase reduces CO_2_ to CO. Furthermore, hydrocarbon chains can be produced, either, by replacing H with D in the buffers or by using the strong reductant [Eu(II)‐DTPA].

### 3.3 CO_2_ reduction by the Fe‐only nitrogenase

The reduction of CO_2_ by the Fe‐only nitrogenase has been characterized in AnfH/ATP‐dependent *in vitro* assays and electrochemically, using a polymer‐coated electrode containing the FeFe protein and relying on cobaltocene as electron mediator.^[74]^ In the AnfH and the electrochemically driven reaction, 31 % or 32 % of the electron flux is directed towards the reduction of CO_2_ to HCOO^−^, respectively. For the AnfH/ATP‐driven *in vitro* assays, the formation of trace amounts of CH_4_ were reported, but the possible formation of CO was not mentioned.

The CO_2_ reduction activity by the Fe‐only nitrogenase was also confirmed in non‐growing *R. palustris*. In contrast to the Mo and the V nitrogenase, strains expressing the wild type Fe‐only nitrogenase formed already elevated amounts of CH_4_.^[26]^ Surprisingly, introducing the double mutations of the V nitrogenase to the Fe‐only nitrogenase (V57A and H180Q in AnfD) stopped the formation of CH_4_ in *R. palustris*.

In a nutshell, the WT Fe‐only nitrogenase has the highest inherent activity for the reduction of CO_2_. Remarkably, the same portion of electrons (31 %) are used either for the reduction of N_2_ or for the reduction of CO_2_ under the according atmosphere.

### 3.4 CO_2_ reduction by the Fe protein

In 2017, Yu and co‐workers demonstrated that the Fe proteins of the Mo and V nitrogenase (NifH and VnfH) are capable of reducing CO_2_ to CO.^[79]^ Sub‐stoichiometric amounts of CO were formed in *in vitro* CO_2_ reduction assays that used dithionite to reduce the enzymes [Fe_4_S_4_] cluster to the [Fe_4_S_4_]^1+^ state. The produced amounts of CO were increased for VnfH compared to NifH, which is consistent with the lower reduction potential of VnfH compared to NifH. The activity was further improved by adding ATP to the Fe proteins, as the nucleotide‐bound states of both proteins exhibit lower reduction potentials. Turnover numbers of up to ∼8 were achieved for the CO_2_ reduction to CO by using the reductant [Eu(II)‐DTPA] in repeated additions. [Eu(II)‐DTPA] reduces the [Fe_4_S_4_] cluster to the all‐ferrous state ([Fe_4_S_4_]^0^). The Fe proteins can also catalyze the reverse reaction, the oxidation of CO to CO_2_, when the reduced [Fe_4_S_4_]^+^ cluster was reoxidized to the [Fe_4_S_4_]^2+^ state by an excess of indigo disulfonate. This suggests a potential involvement of all available [Fe_4_S_4_] cluster redox states of the Fe proteins ([Fe_4_S_4_]^2+^, [Fe_4_S_4_]^1+^, [Fe_4_S_4_]^0^) in the CO_2_ reduction and establishes the Fe protein as an enzymatic mimic of the nickel‐iron CO dehydrogenase, the only other enzyme known to interconvert CO_2_ and CO. Moreover, the CO_2_ reduction to CO was demonstrated *in vivo* in *A. vinelandii* strains expressing only the Fe protein of the Mo or V nitrogenase. After 14 h under 40 % CO_2_, the maximum turnover numbers of 140 and 110 were reported for VnfH and NifH, respectively. These turnover numbers indicate the formation of the all‐ferrous state *in vivo*.

The reduction of CO_2_ by Fe proteins was further investigated for the NifH analogue of *Methanosarcina acetivorans* (NifH^*Ma*^). This organism is naturally adapted to the CO_2_ reduction and occurs in habitats with high CO_2_ concentrations.^[80]^ As expected, NifH^*Ma*^ reduces CO_2_ to CO under [Eu(II)‐DTPA]. However, the amount of formed CO by NifH^*Ma*^ decreased for concentrations above 20 mM [Eu(II)‐DTPA]. Instead of forming CO, electrons were redirected into the formation of CH_4_, C_2_H_4_, C_2_H_6_, C_3_H_6_ and C_3_H_8_. In contrast, the activity of NifH from *A. vinelandii* (NifH^*Av*^) for the reduction of CO_2_ to CO was enhanced by increasing the concentration of [Eu(II)‐DTPA] from 10 mM to 100 mM, no hydrocarbons were detected. NifH^*Av*^ and NifH^*Ma*^ were also tested for their capability to reduce CO. While NifH^*Av*^ shows no catalytic activity, NifH^*Ma*^ formed C_1≤4_ hydrocarbons with a similar product distribution as observed for the CO_2_ reduction. Also the hydrocarbon formation from CO increased with higher [Eu(II)‐DTPA] concentrations. The differences in the CO_2_ reduction between NifH^*Av*^ and NifH^*Ma*^ likely originate from their different affinities towards CO. Under dithionite NifH^*Ma*^ can reduce CO to hydrocarbons at low yields, which emphasizes a potential physiological relevance of this reaction.

**Figure 4:**
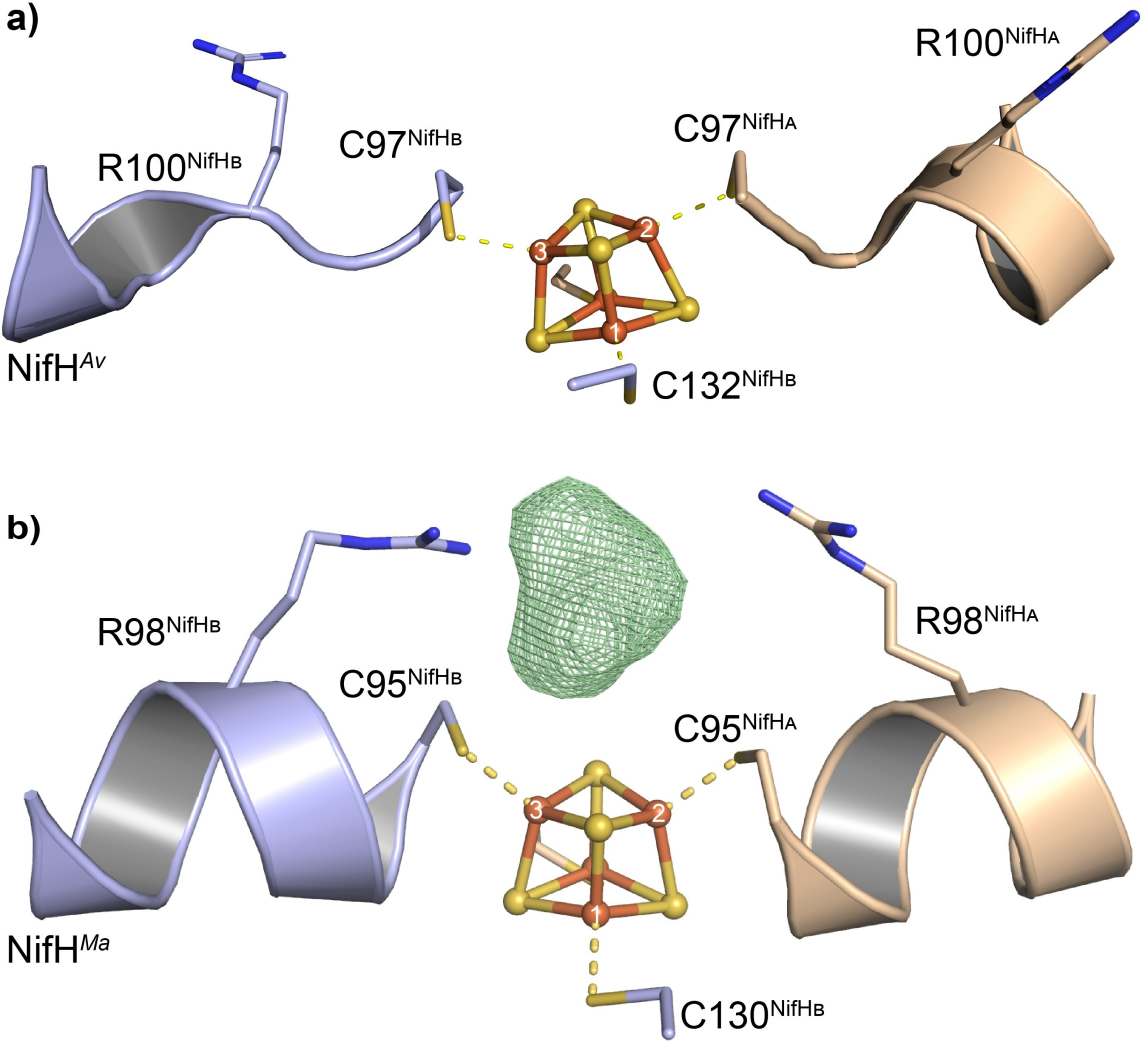
Structure of the dithionite reduced a) NifH^*Av*^ (PDB: 1G5P) and b) NifH^*Ma*^ homodimer with the omit map (*F_O_*‐*F_C_*, green mesh) of the bound carbon substrate contoured at 3.0 *σ* (PDB: 6NZJ).^[81]^

Dithionite reduced NifH^*Ma*^ ([Fe_4_S_4_]^1+^ state) was crystallized in the presence of the alternative CO_2_ source bicarbonate. A 2.4 Å crystal structure was obtained, leading to mechanistic insights into the CO_2_ reduction by the Fe proteins (Figure 4b).^[81]^ The residue R98 of NifH^Ma^ (corresponding to R100 of NifH^*Av*^) is highly conserved among Fe proteins. While the R100 residues in the dithionite reduced structure of NifH^*Av*^ are facing away from the [Fe_4_S_4_] cluster (Figure 4a), the R98 residues of NifH^*Ma*^ are directed towards the [Fe_4_S_4_] cluster in the interface of the NifH^*Ma*^ homodimer in the presence of CO_2_ (Figure 4b). In close proximity to the [Fe_4_S_4_] cluster and the two R98 residues remains additional electron density in the structure of NifH^*Ma*^ incubated with CO_2_, which is unaccounted for by the protein structure. This density could be modelled as a linear CO_2_ molecule, though other ligands like carbonate and glycerol also resulted in reasonable structural models. Further support for the binding of CO_2_ in the crystal structure of NifH^*Ma*^ was obtained by quantum chemical calculations. It was proposed that residue R98^B^ has a role as a proton donor during the CO_2_ reduction and breaks the symmetry of the [Fe_4_S_4_] cluster to facilitate the binding of CO_2_ to a distinct Fe atom of the otherwise symmetric [Fe_4_S_4_] cluster. The mutation of R98 to H98 or G98 showed a decrease in activity of 20 % and 80 %, respectively, supporting the hypothesis that R98 serves as a proton donor. The mechanism of CO_2_ reduction by the all‐ferrous state of NifH was further investigated by DFT calculations.^[82]^ The structure optimization led to a minimum with an activated CO_2_ in a carboxylate‐like conformation bound to the Fe(3) of the [Fe_4_S_4_] cluster, with the R100^B^ interacting with the bound CO_2_ via a hydrogen bond. On the basis of DFT calculations a mechanism was proposed. CO_2_ is guided by R100^B^ to the Fe(3) of the [Fe_4_S_4_] cluster and subsequently activated. The carboxylate intermediate is then protonated by R100^B^ or by the buffer. Subsequent electron uptake and donation of the [Fe_4_S_4_] cluster to the carboxy‐group results in the formation of H_2_O and a CO bound intermediate. The CO is then released and the [Fe_4_S_4_]^1+^ cluster reduced back to its [Fe_4_S_4_]^0^ state by [Eu(II)‐DTPA] to close the catalytic cycle.

Moreover, it was shown that the CO_2_ reduction activity of the Fe proteins is an inherent property of [Fe_4_S_4_] clusters by catalyzing the CO_2_ reduction with the synthetic model compound [PPh_4_]_2_[Fe_4_S_4_(SCH_2_CH_2_OH)_4_].^[80]^ The CO_2_ and CO reduction activity of biogenic and synthetic [Fe_4_S_4_] clusters renders them an interesting research field for the development of new homogenous CO_2_ reduction catalysts.^[83]^

## 4 Biological Relevance of the Nitrogenase CO and CO_2_ Reduction

Organisms that grow anaerobically encounter the problem of accumulating abundant amounts of reducing equivalents in ferredoxins and flavodoxins, if their carbon source is more reduced than their biomass. The enrichment of reduced redox cofactors ultimately prevents growth, if the organism cannot transfer the electrons to an electron acceptor. Three viable mechanisms have been identified in purple non‐sulfur bacteria to independently support anaerobic phototrophic growth from such carbon sources. In the CBB cycle electrons are accepted by CO_2_, DMSO reductases use excess electrons to reduce DMSO, and nitrogenases use electrons for N_2_ and H^+^ reduction and the formation of NH_3_ and H_2_.[^76^, ^84^] Therefore, nitrogenases serve as redox mediators. In accordance with this additional role of nitrogenases, the H_2_ formation by the Mo nitrogenase is increased *in vivo* upon interruption of the CBB cycle by deletion of RuBisCO.^[85]^ The reduction of CO and CO_2_ by the alternative nitrogenases potentially contributes to this mechanism of redox cofactor recycling.

As discussed above, the Mo nitrogenase is strongly inhibited by CO, even under environmental concentrations. To protect the Mo nitrogenase from CO, some diazotrophs express the protein CowN. CowN interacts with the Mo nitrogenase and prevents its inhibition by CO, without decreasing the nitrogenase activity for N_2_ and H^+^ reduction.^[86]^ For instance, the expression of CowN allows the Mo nitrogenase dependent growth of *Rhodobacter capsulatus* for CO concentrations of up to 3.5 %. However, the deletion of CowN did not affect the cell growth sustained by the Fe‐only nitrogenase under CO. Interestingly, AnfA, the transcriptional activator of the Fe‐only nitrogenase, represses the expression of CowN, indicating that the CO protection is not required for the Fe‐only nitrogenase. Therefore, the promiscuous activity for CO reduction by the V and Fe‐only nitrogenase could indicate a function of these nitrogenases as detoxifying enzymes. The amount of CH_4_ formed from CO_2_ by the Fe‐only nitrogenase in *R. palustris* was also sufficient to support the growth of a co‐cultivated methanotroph *Methylomonas* sp. LW13.[^8^, ^9b^] A methanotroph living of the CH_4_ formed by nitrogenases implies that the CO and CO_2_ reduction might play a role in the symbiosis of microbial communities, and not only presents a detoxifying function. Our understanding regarding the biological role of the nitrogenases‘ ‘side reactivities’ is very fragmented and calls for further investigation.

## 5 Summary and Outlook

The *in vivo* reduction of N_2_, CO and CO_2_ by the WT V and Fe‐only nitrogenases implies a potential evolutionary link between the nitrogen and carbon cycle on earth.^[65]^ The need of all living organisms for an N source has selected nitrogenase ancestors specialized for N_2_ reduction. Repurposing the nitrogenase machinery for the reduction of CO and CO_2_ to form C−C bonds is an exciting path to biotechnological processes and the synthesis of value‐added products. Recently, V nitrogenase expressing *A. vinelandii* cells were explored for the production of C_2_H_4_ from CO in 2 l bioreactors.^[87]^ Here, the achieved C_2_H_4_ productivity could not compete with industrial methods. Still, this process can be improved by changing the reaction conditions or by protein engineering. The effect of protein engineering was highlighted by introducing double mutations to the MoFe (V75 A and H201Q) and VFe (V57A and H180Q) proteins, increasing the CO_2_ reduction and CH_4_ formation activity.^[8]^ Naturally, the Fe‐only nitrogenase directs a large portion (31 %) of its electron flux towards the reduction of CO_2_ and is capable to produce CH_4_ inside cells. This activity renders the Fe‐only nitrogenase an ideal basis for the development of reduction catalysts for the utilization of the ubiquitous greenhouse gas CO_2_.

## Conflict of interest

The authors declare no conflict of interest.

## Biographical Information

*Niels N. Oehlmann obtained his BS and MS degrees in Chemistry at the Goethe‐Universität Frankfurt. In 2020, he joined the research group of Dr. Johannes G. Rebelein at the Max Planck Institute for Terrestrial Microbiology for his Ph.D. studies. His research focuses on the conversion of carbon containing substrates by nitrogenases. Currently, he is a Kekulé fellow of the Fonds der Chemischen Industrie*.

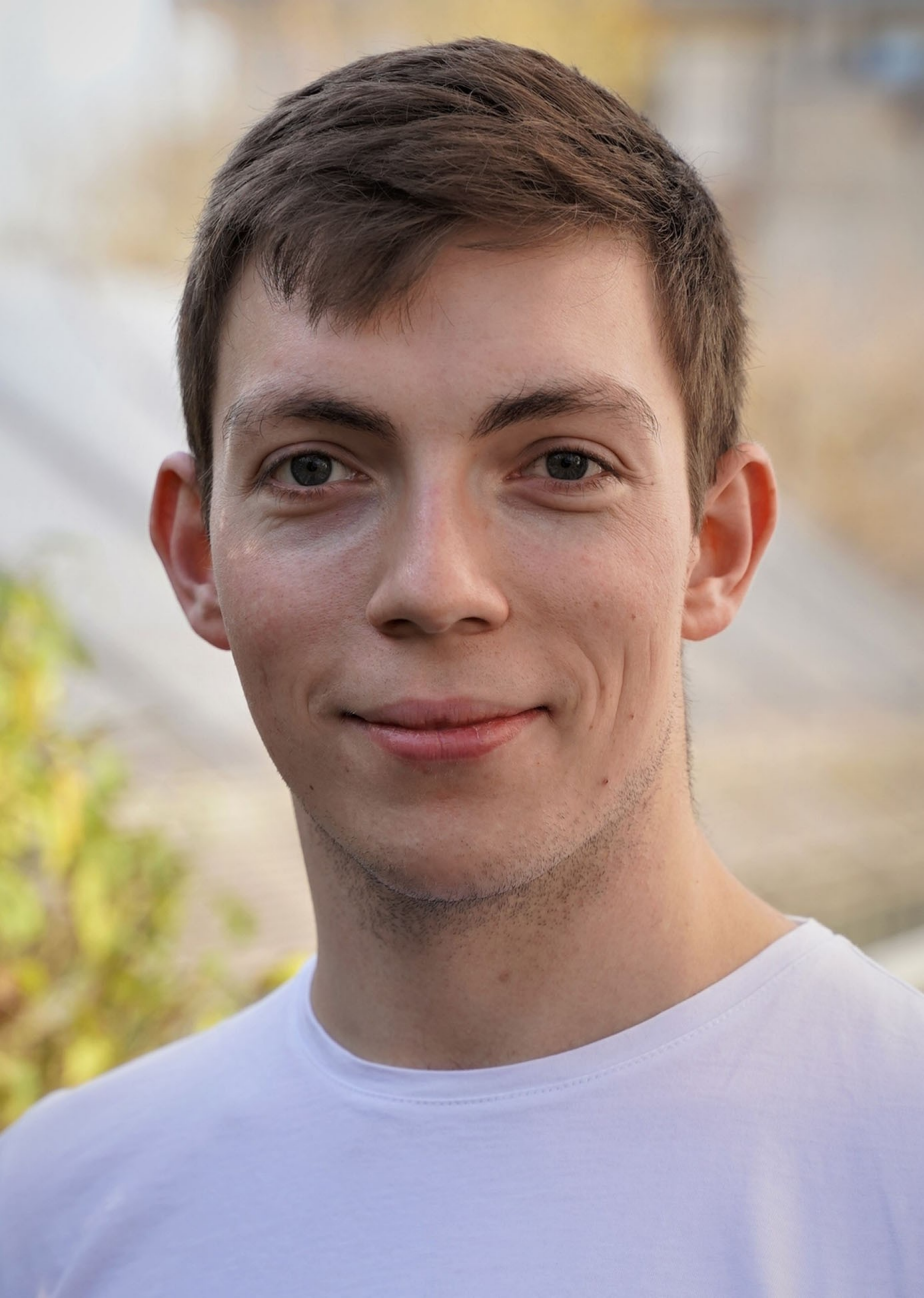

## Biographical Information

*Johannes G. Rebelein received his BS and MS degrees in Biotechnology from the TU Braunschweig. In 2016, he received his PhD degree from the University of California in Irvine. During his postdoctoral stay at the University of Basel, he worked as an EMBO long‐term fellow on the construction of artificial metalloenzymes. He has been leading an Emmy‐Noether research group since 2020, at the Max Planck Institute for Terrestrial Microbiology*.